# Public Standing of the Physician

**Published:** 1901-05

**Authors:** 


### Public Standing of the Physician.

---

[Following is the address of Dr. B. E. Hadra, President of the Texas State Medical Association at the Galveston meeting, April 23-27. His subject was, "The Public Standing of the Physician in Comparison with That of the Other Learned Professions, Especially as Applied to the United States."]

The natural desire of the medical profession to keep the closest contact, and to have the most mutual understanding with all other parts of society, prompted the Medical Association to charge its president with the very lofty, but no less difficult, task to address a general audience on some topic connected with our professional work, and interesting to those who may honor us with their presence. This, then, is the excuse for my appearance before you.

Expecting, perhaps, to be listened to by parents who have to select an occupation for their son, or perhaps by a young lady who has good reasons to weigh the pros and contras of the different callings of young men, I chose for my subject a discussion of the social standing of the physician compared with that of the members of other so-called learned professions. I intend to view this subject in two aspects. I will consider the physician as a scientist, in his great public work, as contributor to human knowledge and welfare; and then also in his more modest, practical occupation of administering relief and health to the individual. It is very difficult, though, to draw a strict line between the two, the scientist and the practitioner, for their occupations overlap each other very greatly. Nevertheless, it can be easily understood that there is a difference between a man who gives his time to purely scientific investigation, devoting himself entirely or at least mostly to experimental and theoretic studies, and the practitioner who to the sufferer applies the results found by the former.

But before examining the scientist's relative standing it would be well to picture to you his elevated, lofty and all-embracing work. It is true that there is no such a thing as an independent self-limited branch of science; that all the different parts of it are intimately connected with each other, that they all have the same object: to find the truth, or, in other words, the laws of nature. Still, there are none of the divisions, as they have been differently named for convenience sake, so central, so ostensibly reaching out into every other one, as medicine, the science of nature's laws in their relation to the human body and mind. You may smile incredulously at the statement that the physician has to be a student of history and geography, but nevertheless it is so, when he tries to clear up the causes of disease due to the habits and laws of old and modern people. He has to look into botany, zoology and mineralogy, to study human evolution and the dependence between organic and inorganic beings. He has to dive into the depths of chemistry and physics, he has to know the laws of light, of electricity; in short, he has to scrutinize every mystery of the universe.

Such is the infinite field of his researches, and it is as you can imagine, such an inconceivably wide field that no mind can grasp even the mere outlines. There are, therefore, hundreds of special studies in his department, named differently, but all parts of medicine.

No doubt, the highest mental quality and activity is necessary for doing the best scientific work in any branch of investigation. It is silly to compare the absolute superiority of one scientific application over the other. It is useless and barren to compare the absolute greatness of the philosopher with that of the chemist, the greatest lawyer with the greatest electrician. They all must necessarily have reached the highest possible development of mental capacity and they all must have labored to their fullest ability.

But it is entirely another question whether they all will catch the people's eye and admiration to the same extent, and whether history will give them all seats in the same row. It is also unnecessary to state that that is not so. And it is to this problem, and principally as much as it pertains to the medical genius, that I invite your attention.

With the deepest humiliation must I state that even the most moderate expectation of the doctor will be disappointed. There is no science to whose disciples so little glory is accorded by the people and by general history. Still, it is futile to accuse the world of injustice. There must be good reason for this fact like for every other, so much more as it has held its place for centuries. If we look through old and middle age history we find not one medical man whose name has become a household word, who has been known by old and young, rich and poor, educated or crude, of any one nation, much less through all the civilized world. No one doctor has become a historical figure. Whilst every school boy knows of a Solon or Alexander, a Socrates, a Homer, a Caesar, Shakespeare or Luther, and many other immortals in almost every branch of mental activity, no medical man has through the thousands of years reached a similar grade of popularity. It has changed greatly in modern times, that is true, and I will take occasion to speak of that later on. To avoid, however, being accused of personal caprice or misconception, let me cite two contemporary proofs out of a good many which could be produced:

First, there is a very interesting "Study of British Genius," published by the *Popular Science Monthly* (beginning February, 1901), where a reviewer of the dictionary of National Biography compiles 859 names of Englishmen of "high degree of intellectual eminence." You see it is quite a goodly number; still amongst that great army the reviewer can find only seven names of doctors. Say seven doctors who through all the time from the beginning of English history to the end of the Nineteenth Century are worthy, as he says, of being considered men of intellectual eminence. It is superfluous to say that an expert can with ease add several dozens of names of great English physicians and surgeons, and it has to be stated, too, that the author robbed our profession of many of its best men by classifying them under other headings. However, it shows how little the world cares for doctors.

The second point is one of our own fabric; it is eminently American. The New York University concluded a short time ago to erect a Hall of Fame to the commemoration of the greatest and most meritorious native born sons of the United States. The executors of this noble scheme selected very carefully 100 electors from the ranks of the foremost scholars of the country, and they, by majority vote, named the men to be so honored. The result was the election of 29 giants; but alas, there is not a single physician among them. Still more, in connection with this a New York newspaper requested its readers to submit lists of those whose names they deemed worthy of a place within that Hall of Fame. There were sent in 715 lists, each containing 50 names, and among them all there was not that of a single member of the medical profession.

What, then, battles against a more generous appreciation of the medical genius? In the first place, it is the open effect of a deed or its equivalent, the manifest usefulness or the direct applicability of a discovery or a new idea, and finally the massiveness of effect that will impress upon the perception of the people most strongly.

The greatest mental performance, whose import and effect may revolutionize all knowledge, will cause little immediate stir except in the narrow circle of experts, and may never or only very late be appreciated by the laity. It makes a great difference whether a man invents a cotton picking machine or whether he detects a new law in the conduction of the nerve power. It is but natural that the admiration of the masses follows deeds which can be easily comprehended and the reverse. For such reason a general or statesman has the best chance to live in the world's memory and to have his name nailed to the wall of the Temple of Fame. Indeed, it is interesting to find how many more men of history owe their well merited glory rather to good common sense than to extraordinary learning and scientific achievements; and it is well that it is so.

Thus the medical scientist, like the student of many other branches of science, has a very poor prospect to acquire public fame, as highly as he may be admired by his fellow laborer. Even under exceptional circumstances, when the result of scientific research may have led immediately to manifest blessings to the whole human family, perceptible even to the most ignorant (think for instance of the discovery of the cholera bacillus and the means of escape from it) even the very nature of such an achievement carries with it the weakness to raise the discovery to the highest pitch of merit in the eye of the masses. For the nature of this work is negative, so to say; the effect of the glorious discovery is mostly preventive. It does not impress itself so forcibly upon the average mind as a positive discovery which adds to earthly commodities. It is too much to ask of the ordinary man to rejoice in the removal of a danger which does not threaten in open daylight and to keep continually in grateful commemoration the gallant man who killed a tiger which perhaps would never have made an attack. Thus in a few decades Asiatic cholera, to use the same example, may be known only as a queer reminiscence found in the story books of civilized nations, and you will not suppose that the great discoverer's name will hold its place of glory perpetually. His name will long have vanished from the people's almanac when Robin Hood will still be a celebrated figure in song and play.

Another point. It is a plain fact that, as unexplainable as it appears, the love for possession is more forcible with the masses than the fear of disease, or even of death. Men will live in the most deadly swamps and in the most barren deserts if they can amass a fortune. And not only individuals, but whole nations show their readiness to exchange the health and life of their people for material advantages. Increased care for one's own health by provident self-protection is the attribute of high culture, and the two are proportionate to each other. The crude uncivilized peasant will care much more for his cows than for his own health. No wonder then that medical science and its students stand a poor chance for fame with the great bulk of mankind.

Still, ladies and gentlemen, all these arguments do not suffice to explain the medical scientists' surprisingly low relative standing in life and history, but not to exhaust your patience before my store of provisions I will ask you to listen to only one argument.

If we look with unbiased candor at the history of medicine we must concede that as a science she is a very young child of human mentality. We have no right to call a sum of scattered incoherent bits of knowledge, stuffed and invested with arbitrary speculations, greatly based on superstition and bigotry, by the supreme name of science. But such was the medicine of old. And almost up to the beginning of the nineteenth century the art of curing was directed to the individual only and mostly from case to case in a very contracted narrow circle. There were wholly missing great general principles based upon investigation and knowledge. Medicine had not risen to the position of the great benefactor of whole peoples of the whole human race; she was totally unable to wrestle with the horrors of endemics and epidemics to which whole nations succumbed. A helpless helper stood the doctor by the perishing masses. What, then, should have made him the acknowledged hero of the nation? As much as the single individual may have adored him he had no chance to become the great man before the world and before history. It is therefore not surprising if we find no medical name in the annals of perennial fame before our era. But how wonderfully that has changed. What a proud, splendid edifice has human ingenuity erected in the last century. Physiology, pathology, biology, bacteriology and how all those pillars of eternal brilliancy are named have made medicine the strongest bulwark of human welfare! Were it not that we are dazzled by the immenseness and variety of modern discoveries, the medical portion of them would suffice to call this period of history the blessed and enlightened one. Think what we have learned: Cholera, diphtheria, typhoid, malaria and yellow fever and many more scourges of mankind have become mere scarecrows. Think that we have learned to make accessible to the repairing hand almost every nook and corner of the human body! And should there be no one of the giant minds which have led in this glorious work worthy of the perennial gratefulness of man? Should not one of the men who have saved hundreds of thousands of lives be entitled to a place in the Hall of Fame as much as he who killed thousands in battle? Well I think that no sane person will question the righteousness of our claim, and I think the time for recognition is already at hand. I do not doubt that any of the old nations which had to select but ten of her best men would not fail to include at least one of its medical giants. So much the more it seems to me we owe to the medical profession and not less to the whole country an investigation into the causes of the surprising results of that New York university election.

There are as a whole three classes of great men to be considered: 1. Such as have won the highest pitch of honor in public service as statesmen and generals. 2. Those who excelled in art and literature. 3. Men of science in the widest sense.

If we be true to ourselves, we will confess that only in that first class the full measure of a world record will be conceded to the people of the United States. It is only there where we produce men equal to the best and greatest history knows. A Washington, a Lincoln, a Grant, a Lee, will hold their places at the top of the list of the great statesmen and generals of all nations and all times. But how is it with the other classes? It would be idle vanity to claim that our nation has produced a Michael Angelo, a Beethoven, a Dante, a Moliere, a Goethe, a Shakespeare or a similarly sublime mind wonder. We have creditable, noble and excellent artists and writers—who would deny it? But really great men whose deeds have made an epoch in the path of human development or brought about a revolution of thoughts and sentiments we have not. There are many who are a yard and a hundred yards, but none who are miles ahead of them.

And now as to science. The same is true here. We have not produced a Copernicus, a Newton, a Darwin, a Helmholtz, or a Spinoza. There are a great number of extraordinary bright, ingenious men of the greatest inventive power and of the most striking capacity for developing given ideas, but there are none who have opened new roads by scientific research. And this is certainly also true as to medicine. There has no epoch-making mind appeared in medical science in the United States; though we have as eminent practitioners, as eminent surgeons as any country, full of genius and daring. The contributions to our art are plentiful and valuable. We have to boast of having applied anesthetics successfully the first time, we can boast of the first man who performed such an important operation as ovariotomy; we have a Marion Sims, a marvel of technical ingenuity, but we have no one who caused the medical world to change its thoughts and views; no man who made an epoch or founded a so-called new school, if we except the wonderful revelation of Christian Science, osteopathy or the like. Thus the medical branch is neither worse nor better off than other branches of science in our country. And though we can not offer candidates of the caliber of a Jenner, a Lister, a Pasteur, a Virchow or a Koch, we are, I think, entitled to the same consideration as all other sciences.

I am afraid that the physician's work in our country is viewed too much as a mercenary pursuit which the world does not feel like honoring by triumphal arches. The intimate connection with practical application of the results of our science makes people get the wrong idea. They see no more in medical progress than in that connected with making shoes, perhaps, or any other similar affair, intended to accommodate the workman rather than the people.

Yet, ladies and gentlemen, the point of far more general importance is why the United States has not produced the highest type of the great man of science.

Let us then devote a few words to an attempt to explain this depressing fact to ourselves.

It is evident that a new country, a new nation and a new society will first have to look for the foundation of its material existence. Food and shelter, life and freedom, are the first objects of public thought and action, very naturally. Consequently all brain and muscle will be set to work for this purpose, and the positions of statesmen or military men will be the most prominent and most desirable. Art and science have to stand back until the conditions have been well settled. We know, though, that this period has passed, and that we today rank with the most progressive and cultured nations of the world.

A second cause for the backwardness of our country is the want of government help for institutions and schools. While in the old country it is one of their foremost cares to foster art and science by appropriate support, it is left here too much to private enterprise and private love. Only lately have State governments begun to provide for higher education. I do not want to enter into a dispute as to which system will in the end lead to better results—paternalism or individualism. Besides, by offering well founded positions promising a progressive income for life, the government of the old countries induce great numbers of studious young men to devote themselves exclusively to scientific and art studies, freed from that fear of starving if they should not accidentally find a byway to procure bread and butter. We have nothing of that kind, and consequenlty he who has to elect his career will look to a more or less commercial occupation to be sure of a livelihood. Our profession is the latter, at least half commercial, and there are still fewer places to be had for those few, and no sensible man will figure on them.

These are some causes of our lack of great stars in art and science, but I am almost sure that they are not the most potent ones. The real cause I see in the methods of our schools, which have only recently begun to change.

The method of teaching dictates the quality and direction of the mental work of a nation. Now, there can be no doubt that there is no people on earth with such a high average education and intelligence as ours.

Our late great statesman, Blaine, put the question, "What is preferable: to have such a high level of general education with a generous number of not too exceedingly high towers; or, rather a low general level with unproportionately immense elevations in a few?" I do not want to solve this proposition, except as to science. In this relation, though, it seems but natural that only the highest scientific education will produce the highest scientists, and that such a one, as a matter of course, will be more apt to make great strides forward than many lesser lights combined.

Now, then, our educational methods in connection with the extensive self-education by reading newspapers, produce this general high level of common knowledge, but they are inimical to the production of an aptitude for the very highest scientific applications. The great minds we want are products of a steady organic evolution; they do not fall from the skies, and they do not turn up unconnectedly with the past. They must, aside from possessing inborn excellence of brain, be carefully and steadily trained in the power of concise observation and of logical thinking. The time has gone when lumps of gold were found on the street. Mankind now has to dig deep and long into the mountains and fly miles high into the air to find a grain of knowledge.

The very excusable and comprehensible desire of the individual to get commercial independence calls for schools which offer pre-eminently two advantages: quickness, and as great an amount of positive knowledge as possible. But this again means the acquaintance with only the results of scientific research. Such schools do not make their pupils investigators of the sources of things or detectors of nature's laws.

There is a great mass of such intelligence given by public and private schools, and they are vieing with each other as to rapidity and quantity. They and the newspapers crowd knowledge into the brains of children and adults. But they make no logical thinkers, no minds trained for those highest mental tasks, for correct and intrinsic observation, comparison and conclusion. If one or two pre-eminently adapted subjects would be taught in that painstaking, slowly growing, thorough way as, for instance, Latin and

Greek have been treated in the old classical school, the mind would become so pliable, so impressive and comprehensive, that the facts in all other branches of learning would either alongside or afterwards be taken in with much greater ease and facility. Those who do not pay sufficient attention to this point laugh at the idea of beginning the study of Latin at eight or ten years of age. A two years' course in later life seems enough to get all they want; that means a number of vocables and a fair reading faculty of Latin writings. We forget that it is not for that purpose that this language is taught. And I may say here in parenthesis that I am not one-sided enough to insist on Latin; any other study may do as well as long as it is adapted to the purpose of training our logical power. Outside of this advantage the old-fashioned and, fortunately, in our country, more and more revived method cultivates another feature; that is, training the attention to the small and smallest matters of a subject. It is a practical adaptation of the true proverb that a hundred small things make one great one. Gaps in scientific understanding are only in this way avoided and the basis for continuous chains of ideas formed. The systematic development which necessarily must be slow, like every organic growth, is the only safe way to produce great minds. Steady, toilsome work is half the genius, as a genius himself put it. If I am in favor of the so-called classical training, I am so because I am convinced of the value of that heavenly perfume emanating from the work of the old founders of our civilization. It seems to me the finishment of that subtle refinement which is the most appropriate toilette of the really great.

Now no sane man will doubt that the American mind is equal to that of the most cultured peoples of the Old World. And we will surely produce as many and as great men in the future as they did and do. We only need conditions and schooling better adapted to this end. Up to that we will continue to issue a great number of eminent men, you may say the best; men who will reach high distinction and prominence by helping to develop new ideas. The American has proved to be the greatest inventor standing upon ground prepared by others, but he will soon begin to prepare his ground himself, and then there will be no lack of men to be honored by statues and busts in the Hall of Fame, neither will the doctor of medicine be wanting among them.

Ladies and gentlemen, your interest in my subject will, I think, greatly increase if we enter the investigation of the standing of the practical physician, of the doctor, in comparison with that of the members of all other so-called features of social and political life. It will be a matter much nearer to you, to your affections and your sentiments.

It goes without saying that the moneyed man, the man in commercial pursuits, may be as much learned and as much accomplished as the professional man. And in many aspects of public life he will be the natural leader. Wealth and its influence will make even the crude man a very important factor of human affairs. But there is a sphere in which the professional man will not by necessity, but by a kind of general consent, be the representative of society; and I repeat, it is not the individual I am going to examine, but the class, and there are many exceptions one way or the other, that is understood. The high position of the learned profession is ostensibly due to the higher mental qualities, or say to the greater ability to grasp and treat man and the thousands of events and situations of human life. Among the different professional men the difference in such quality will decide their standing. The more adapted one will be the more will he be intrusted with leadership.

Comparing, then, the most conspicuous learned avocations in their relation to practical joint affairs of life, we may first mention the minister of the gospel. Aside from the traditional restrictions of his position he will be hampered and handicapped by the cramped border line of his mental field. He is not free and independent, his duty is to stand on the dogma of his creed, and his speculations must start from this basis. As he ceases to do so he ceases to be a minister of his church, and he becomes a moral philosopher. This limitation unfits him as a class for the unbiased adaptation of his thoughts and opinions to the immensely varied interests and aspects of public events. He in everything will be more or less a partisan. All this I say with due respect for this avocation and its members; nothing is farther from me than to cast the slightest reflection on their high calling.

Considering next the teacher, the philologist and the philosopher, it would be absurd to charge them with a want of capacity. But it is the outcome or at least an almost unavoidable feature of their methods of thinking that they become doctrinair and least yielding to other people's views. They become pedantic and adapt themselves only with difficulty to the ordinary affairs of society.

Looking at the learned men whose business is mostly the handling of things of concrete and material nature, the druggist, the engineer, the architect, the electrician and so, he will be mostly too realistic to look at less tangible things in a desirable pliability. He will be too much matter of fact man; he will view affairs more like inorganic objects.

So there remain two professional classes to be investigated, the doctors and the lawyers. And here we have arrived at realities; they are in fact the rivals in the leadership of social affairs of secular nature.

At the first glance it appears as if the doctor could not help but be the competitor destined for victory. He is the man who dives into the most intimate and most various events of humanity; he who is in constant contact with rich and poor, with old and young; he the confidant of everybody; he who learns to know the wants of the human heart and brain; he who has to keep abreast with every new turn of public feelings and doings—I say he ought to be the right man. And still it is not so. It is the lawyer who represents the town, the city and the State; he who makes a speech when a corner stone is laid, a celebrated guest is received, whenever a railroad enters a place, whenever a new enterprise is to be inaugurated; it is the lawyer who opens public gatherings, who presides over them; the lawyer who is the most conspicuous candidate for the mayorship; a lawyer who is sent to the legislature, to congress; a lawyer who is made minister of war, of the navy, of the interior, of agriculture, and a lawyer, as almost a matter of course, has to be the national president. Think of the idea that a doctor run for the presidency. The mere mentioning would be considered farcical. Yet in other countries there have been merchants and engineers and doctors who have ably filled such exalted positions. It is only here that the belief prevails that it must be a lawyer.

We can easily see that the doctor's personal or commercial interest will make him rather abstain from extensive employment interfering more or less with his business. We can also see that the comparative smallness of his professional money transactions may reduce the dignity of his calling; perhaps also the devoted manner toward everybody, very often that of the retail grocer, lowers him in the eyes of the masses. Then the enormous number of quacks and ignorant smarties, charlatans and mountebanks who infest our ranks and disgrace them by their uncouth methods must necessarily offend the sense of the refined and cultured part of mankind. The facility with which superficial medical knowledge may be gotten or simulated, the difficulty of the public to discern the learned and honorable doctor from the disreputable pretender, the seeming ease with which the proverbial old woman may compete with the learned doctor, and not the least the obvious mistakes and errors which even the best must occasionally commit as the natural consequence of the imperfectness of human knowledge—all these factors help to make our profession appear much less dignified and less entitled to a very high valuation. It is true that some similar difficulties are encountered by the profession of law, but they are certainly not so conspicuous and have not such a debasing appearance.

Then the lawyer has the great advantage of being much more expert in public speaking and more skillful in catching the hearers' whim and taste. This, no doubt, is a great item.

But all these points seem insufficient to explain the preponderance in public life of the lawyer over the doctor. It is a fact America is governed by the lawyer, not insisting upon the correctness of the ugly saying that he also governs for himself. Even though by and by the banker, and manufacturer, and the speculator has gained an almost tyrannical position in our country, the lawyer defines and forms the terms for their action, he fosters or inhibits it by way of laws made in legislature and congress. What, then, makes the lawyer so powerful in the smallest as in the most important social affairs? There necessarily must be more than the above named external causes at work.

Thirty years ago I came to this country, leaving a land where the destiny of the individual is more or less mapped out in his early childhoood by the governing powers, or at least by rules of custom.

A child dedicated to one of the learned professions, for each of whom there is a strict path designated, had to begin early with the training prescribed, and the so-called classical education was and is considered a necessity for every one who wants to become a lawyer, a preacher, a doctor, a high-class teacher and so on. Yes, even the more humble civil service appointee had to go throguh some such training. As an example, an individual whose highest attainable position is that of postoffice clerk, whose duty is perhaps the delivery of letters at the window, has to show up some knowledge of Latin, perhaps of Greek. Brought up under such impressions, I felt almost convinced that such was the dictate of natural laws. Now imagine how I was startled and shocked by the incredible things which revealed themselves before my gaping eyes. I met governors, statesmen, supreme judges and, in short, the highest possible public officers, of course, every one a lawyer, who I was told had been tailors, or farmers, or ox-cart drivers, or what else before; that one had no school training at all, the other perhaps a few months in a country establishment, but a very few a college education. I considered such information nothing but jokes. But it was the truth, and there are yet living proofs of that truth. Then when I had opportunities to listen to their public speeches of hours' duration, speeches which combined fluency with mental refinement and logical accuracy, proving the fullest grasp of subjects of high order—when I read their messages, their legal opinions, their charges to court, in short, the products of their professional attainments, I confess that I began doubting the value of the supposed higher culture of my old country, and still more I began believing that the brains of the new world were of immensely higher quality. It took me a long time to solve that conundrum to my own satisfaction. I had, however, to dismiss both the mentioned explanations. I could not make myself believe that culture and mental training should go for nothing, neither would I accept the possibility of such a gain in evolution of the brains of the American in two or three generations. It was against all biological deduction and experience. The individual freedom, the unhampered development of the abilities of the individual, the easy change of occupation, so as to find finally the most adapted one after others had been cast aside; the general participation in public affairs, the general reading of newspapers—all that must have had its telling effect, that is evident. But then this striking phenomenon had almost exclusively applied to the lawyer. He constituted the only profession that showed this extraordinary gain. He was beyond doubt the most trained, the most able, the most influential person—in one word, the leader of mankind. He had been the general in war, he was now the man on top in peace; he was the lawgiver, the executive, the judge, in fact the shaper and master of public life. I could not see much of prestige in the doctor's position. He was the modest administrator of drugs and the knife, then and when cutting a prominent figure in society, but then more accidentally, owing to a domineering intellect, or to his personality or wealth. Still, the doctor had a good deal more of professional schooling, he had been in the medical college, in hospitals for years, while the lawyer perhaps never left his little town. As to previous general education, they were certainly on equal footing. What, then was the cause of this divergence? What made the lawyer so predominant? I think I do not err if I solve the problem by conceding to him the better logically trained mind. It is the greater precision of perception, the more logical consideration of things, that makes one man the master over the other, may this quality be a gift of nature, or acquired in daily experience, when it is called good common sense, or mental training. Supposed equal mental conditions when the lawyer and the doctor began their studies, the latter will soon be ahead as a logician. The lawyer, unknowingly, at once begins the exercise of higher school education the first day he reads law. He in the very daily application of his business is continuously at a school of logic, so to say.

He will not overreach only the doctor, but also every other professional man whose business is less of that purely logical nature. Thus he makes up for the deficiency of his schooling very soon, and keeps on developing through all his life, necessarily gaining steadily over all others who do not train the same way. This, too, seems to me the right explanation for the less striking superiority of the lawyer over men of other learned professions in the old world. There the latter receive a much more intensive training of their logical faculties, and consequently the difference between their and the lawyer's thinking ability is smaller.

If this solution of the problem should not seem to you to hold good, I would invite you to compare for a moment the mental employment of the lawyer with that of the doctor. The lawyer has above all to be the most unbiased and cold, and the same time the most scrupulous observer of his subject. The least little giving to sentiment and emotion may turn out disastrous because his opponent will make use of any fissure left unprovided. So he has to be of utmost watchfulness in the most insignificant details. A wrong semicolon, a falsely written name, anything hardly worth noticing in other occupations, may lead to a Waterloo. Then the connectedness of his deductions must be absolutely unbreakable, that means strictly logical; his adversary never will cease to lay in ambush. Further, in his general method of thinking he is not hampered by anything which he can not grasp by his own intellect. Though he has to accept actual law and opinions of the courts, he is nevertheless to himself a competent judge as to the righteousness of these laws. He in one word does not need anything but his own logic as his guide.

And now look at the methods of the doctor's work. He has to take for granted the thousand and one opinions and outcomes of infinitely diverse laboratory researches. He can not go behind what these investigators tell him, because he has neither the time nor the special training to put it to the test. He has to take most on mere faith; and, still worse, the findings of the investigators will ever change, or will be differently explained, they will rarely be undisputable facts. Knowing this and rarely enjoying the happy feeling of conviction, he will fall in the habit of becoming satisfied with probabilities and with only hypothetical correctness, which habit is adverse to logical precision. In fact, medical art is an art of probabilities. As accurate and logical, therefore, as his thinking may be, it will consist of more or less long jumps from one known fact to the other, which he will make in the dark as good as circumstances permit.

And here I would like to make a remark apropos to our investigation. A few months ago the governor of Colorado vetoed a bill regulating the practice of medicine in his State. Between some, as I believe, well taken points there is an argument which shows that even lawyers will not always be sound logicians. He there says: "The development of surgery excepted, medicine is not a science. It is a series of experiments more or less successful, and will become a science when the laws of health and disease are fully ascertained and understood." Now, I can hardly think of a greater misconception of the nature of science, which is, I have said before, investigation, gathering and classification of phenomena in order to get at the laws of nature; it is the search for truth. As soon, then, as a truth has been found it invariably becomes the common good of human knowledge, it ceases to be the object of science. If the governor of Colorado would know all the laws of health and disease he would be a very smart fellow, but not a scientist, and perhaps Mrs. Smith next door would know them as well as he. He would not have to work by "experiments more or less successful," because that would be absolutely superfluous when everything were already known. He would hardly get a position as a professor of medicine in a laboratory, because there would be nothing to search for.

But let us return to our subject. If it is correct, as I contended, that the more developed the logical reasoning of a person becomes the more his power will grow over others, then the public leadership of the masses in our country belongs to the lawyer. The practicing physician has to stand back, that is evidently his fate, unless he trains his mind before he enters his professional career, much more than the lawyer, in order to bring into his business a plus of logical thinking ability; and unless he exercises his mind continually in more abstract studies along with his professional ones.

If I am correct it was Gladstone who once said that the lawyer's reign has come to an end, and that of the doctor's has begun. I do not think he is right about that as far as our country is concerned, at least not for a century to come.

Now, ladies and gentlemen, I would be sorry if I had made the impression upon you of that ugly bird that soils its own nest. Nothing is more remote from my intention. I simply want to give, if you excuse the phrase, the devil his due. There are so many redeeming features in the mental methods and the nature of the intellectual work of the physician that he can afford to be generous. Let me count up a few of his superiorities. His manner of looking and judging is by far quicker and more comprehensive. He must make the combination of the visible and the mostly circumstantial evidences often on the spot. He has no time to reflect and consult his library in most of his cases. Also is his understanding much more educated and trained for the situation as a whole. Say for the totality of the picture. But what I would consider the most glorious point in his favor is the professionally characteristic feature of referring all matter and event, not only his professional affairs, to human life and the human being. That is a perfectly different habit from that of the lawyer whose object is to arrive at the most correct expounding of abstract right and wrong. The medical man is surely the most human and the most humane of all professional men; he who never wavers to kill his own goose that lays the golden egg, when common welfare so demand. You see him daily teach you how to prevent disease, though he lives on its very existence.

Take it all in all, the fields which the two professions cultivate are widely separated. The two are really not rivals in a closer sense. Whilst it belongs to the pursuer of the law to appear before the masses, to make himself conspicuous before the crowd, especially when he enters that byroad of politics; the domain of the doctor is the retired hospital and the sacred privacy of his office or the sick room. The individual and the family he has to befriend and to conquer, and not the masses. He may be better loved by the community, loved by more people than the lawyer, thought more of as a man and a citizen; still he will have to stand back when it comes to the loud public honors of the street and the political arena.

And thus I will close my tedious discourse, hoping that you will think of the doctor, if not better than before, at least not worse.

